# A hybrid approach for forecasting peak expiratory flow rate in asthma patients using combined linear regression and random forest model

**DOI:** 10.1371/journal.pone.0326036

**Published:** 2025-08-21

**Authors:** Shayma Alkobaisi, Wan D. Bae, Muhammad Farhan Safdar, Najah Abed Abu Ali, Sungroul Kim, Choon-Sik Park, Robert Marek Nowak

**Affiliations:** 1 College of Information Technology, United Arab Emirates University, Al Ain, United Arab Emirates; 2 Department of Computer Science, Seattle University, Seattle, Washington, United States of America; 3 Department of ICT Environmental Health System, Graduate School, Soonchunhyang University, Asan, South Korea; 4 Department of Internal Medicine, Soonchunhyang Bucheon Hospital, Bucheon-si, South Korea; 5 Faculty of Electronics and Information Technology, Warsaw University of Technology, Warsaw, Poland; Sreenidhi Institute of Science and Technology, INDIA

## Abstract

Asthma is a frequent and long-lasting disorder associated with airway inflammation. The disease severity may lead to serious health concerns and even mortality. In this work, we propose a novel hybrid approach using machine learning models and similarity measurement technique with the aim of precise peak expiratory flow rate (PEFR) estimation for asthma trigger assessment. The random forest model was first utilized to classify the PEFR percentile zones on unseen data. Then, two linear regression models following thresholds of <50% and >=50% were hypothesized and trained to achieve better outcomes than a single standalone model. Hence, the input is diverted to the relevant model for prediction based on classification results. Furthermore, a string-matching technique has been proposed to obtain reference outcomes in addition to yesterday’s PEFR. Finally, a supplementary linear regression model is used to make predictions based on input of two prediction values and one PEFR value from the previous day. The proposed model is evaluated on a dataset of 25 patients, each with 2 to 3 months of recordings, on average. The findings showed reduced mean and random absolute error of 27.064 L/min and 1.34%, respectively, using the suggested model, compared to 79.794 L/min and 4.42% error rates by the standalone linear regression model on five-fold cross-validation. The outcome indicates that the proposed hybrid algorithm accurately predicts asthma-trigger events.

## 1 Introduction

Asthma is a chronic inflammatory condition of the airways and presents a significant global health challenge, affecting an estimated 262 million individuals worldwide. Despite advances in medical technology and public health initiatives, asthma remained a leading cause of morbidity and mortality, with approximately 461,000 reported deaths in 2019 [1,2]. Asthma triggered by various environmental factors such as dust mites, air pollution, and tobacco smoke. It is characterized by symptoms including coughing, wheezing, and shortness of breath. The complexity of asthma lies in its heterogeneous nature, driven by specific immune cell activation [3]. Diagnosing asthma typically involves a combination of clinical evaluation and spirometry testing. Treatment strategies range from inhaled corticosteroids to personalized approaches tailored to individual patient needs [4–6]. Despite ongoing efforts, there is still a need for further research and innovative approaches to deepen our understanding of asthma, improve its management, and ultimately prevent asthma-related illness and deaths.

The environmental factors are crucial elements that causes onset asthma attacks and involved the development of childhood asthma. These factors consists of weather conditions, exposure to allergens, life style, and use of tobacco [7]. Climate change further increases the environmental challenges, raising the risk of patient exposure directly, such as heatwaves, and indirectly, including air pollution, higher allergen levels, and increased microbial exposure. With the technological revolution, numerous methods have been introduced for detecting, predicting, and diagnosing asthma.

Artificial intelligence (AI) is a technology that follows human intelligence processes through machines and mathematical models. Recent advancements in AI have enabled the development of mathematical models capable of real-time prediction and forecasting of disease exacerbation, e.g., asthma, based on an individual’s data. Broadly, AI can be categorized into supervised, unsupervised, and semi-supervised learning. Machine learning (ML) is a subset of AI that typically requires manual feature extraction during model training, while deep learning (DL) is a more advanced subset of ML that automates the feature extraction process. ML-based models rely on statistical methods, while, DL utilizes neural network architectures with multiple layers, allowing them to handle more complex data. These AI methods find applications across various domains, including healthcare, weather prediction, and business analytics, for numerous tasks such as classification, clustering, and trend forecasting [8–10].

Regression analysis, a common ML approach widely considered in healthcare research problems, predicts the outcome based on dependent and independent variables [11]. These variables, also known as features, hold numerous records learned by the mathematical models in delivering the outcome or predictions. The predictions can be either binary or a time series. During the model training phase, a loss function, such as Huber or quantile loss, plays a crucial role in evaluating the model’s performance, preventing overfitting, providing prediction intervals, and refining the final analysis [12]. Hyperparameters are essential in fine-tuning the model to better fit the data and outcome requirements. Moreover, employing more than one model, often termed a “hybrid model”, can enhance performance. For instance, a review study by Behrang B. et al. [13] explores that ML models, i.e., artificial neural networks, support vector machine, random forest, and feed-forward neural networks for hybrid modeling, were utilized more frequently than other models [14]. Random forest (RF) is an ML algorithm that uses several trees, where each tree is trained on the subset of data, and the decision is made independently. A voting mechanism in the RF algorithm is adopted to reach the final prediction based on the multiple trees majority voting. Likewise, linear regression (LR) is the statistical method that measures the association among independent (*X*) and dependent (*Y*) variables. LR is used to predict the continuous numerical values. The aim is to find a more optimized linear function to determine a set of coefficients that helps predict the dependent variable more accurately [15]. It can be described mathematically as expressed in Eq 1.

$$Y=\beta_{0}+\beta_{1}X_{1}+\beta_{2}X_{2}+\cdots +\beta_{n}X_{n}+\varepsilon$$ (1)

where $\beta_{0}$ denotes the intercept, $\beta_{1}$ to $\beta_{n}$ illustrates coefficients, *X*_1_ to *X*_*n*_ are independent variables and *ε* can be read as error term [15].

### 1.1 Study significance

In this paper, we introduce a novel method that considers a hybrid model for classification and regression for predicting a common asthma risk predictor known as peak expiratory flow rate (PEFR) measurement. First, the percentile zones are classified using the RF model. Then, its output benefits from choosing suitable distributed LR models at layer 1. Further, a string-matching technique has been adopted to obtain reference outcomes in addition to yesterday’s PEFR, which are then input into the auxiliary LR model at layer 2. The proposed algorithm demonstrates superior performance compared to the existing standalone LR, as evidenced by reducing the mean absolute error from 79.794 L/min to 27.064 L/min and relative absolute error from 4.42% to 1.34%, as depicted in Table 5.

The main contributions of this work are summarized as follows:

- The proposed algorithm highlights the significance of RF and multiple LR models alongside similarity matrix evaluation in specific asthma event assessment.
- An arrangement of the presented composite framework enriches the regression task and could be considered in particular healthcare problems.
- Realizing the simple ML models, including RF and LR, can contribute to overcoming memory overhead issues.

The remainder of the paper is organized as follows: Sects 2 and 3 describes the existing work and detailed implementation methodology, respectively. Sect 4 illustrates the improved outcome with a discussion directing to the study’s conclusion.

## 2 Related work

AI algorithms are used in many areas of healthcare to analyze data, including tasks like sorting information into categories, predicting outcomes, grouping similar data, and processing clinical notes. The existing related literature is structured into two sections, with details provided below.

### 2.1 AI in medical data

Logistic regression (LGR) is a classification model that uses the logistic or sigmoid function to measure the probability of belonging to a class presenting an output. One such example is a prediction of pediatric asthma hospitalization in an emergency department by Marion R. et al. [16], where they considered RF and LGR, showing promising output. The electronic health record (EHR) refers to computerized clinical notes, treatment, and laboratory investigation reports used as the dataset in their work. It was expanded over five years in study [16], having prominent variables of prior visit outcome, ESI level, comorbidity, medication time, and individual characteristics. Feature importance score was also figured. They considered the area under the curve (AUC), which is a performance metric used to estimate the model performance where “1” depicts the highest, while “0” is the lowest, showing 0.83 and 0.88 for LGR and RF, respectively. Panagiotis G. et al. [17–19] proposed a novel model named “data ensemble refinement greedy algorithm” (DERGA) considering five ML algorithms including decision trees, extra trees, RF, gradient boost and Gaussian process classification in predicting ICU vs non-ICU hospitalization for the COVID-19 patients. Further, they also predicted the hospitalization and mortality rate for COVID-19 patients with the help of an artificial neural network. The variables included age, gender, and complement genes for the model predictions, showing >90% accuracy [20].

An similar concept was adopted in the work by Iqbal A. et al., where they used the individual voice to classify asthma among healthy individuals. Instead of EHR, their data was voice or cough sound signals, performing time-frequency analysis with the help of a spectrogram. The dataset included 300 samples, including speech, asthma & cough sounds. A classifier based on an evolutionary neural network was used, which attained 99% accuracy classification [21]. The smaller sample size evaluated in their study could introduce bias into the proposed methodology results. A review by Iman R. et al. [22] based on forecasting of COVID-19 also expressed that the compartmental models that refer to the four stages, including susceptible, exposed, infected, recovered, often termed as “SEIR” were applied more than the others, followed by DL techniques.

### 2.2 AI for asthma and environmental predictions

Chronic obstructive pulmonary disease (COPD) is a lung condition that causes restricted airflow and difficulty in breathing. A review of over a decade (2013-2022) of published work was conducted on COPD by Xu S. et al., highlighting the proportion of medical records (16%), medical imaging (22%), genetics (12%), airflow (15%), signal (18%), and miscellaneous (17%) data across their 67 selected studies. The DL techniques outperform ML [23]; however, it is fundamental to understand that DL requires more computational power than ML. Wearable devices with diverse features, including heart health signals, are becoming familiar. Therefore, Rahman J. et al. [24] utilized a chest band and smartwatch to estimate the heart rate variability among healthy, asthma, COPD, and co-morbid patients to categorize the individuals. The highest accuracy of 82.07% was attained from the chest band data using the AdaBoost model.

Particulate matter (PM) is the microscopic particles of liquid or solid suspended in the air and can be inhaled, causing severe health issues. A correlation between PM and PEFR was uncovered by Bhat G. et al. [25]. PEFR is the flow rate measured through forceful exhalation and estimated using a specific device. An IoT-oriented system was used to gather the relevant data from individuals and indoor/outdoor environment. The DL model, popular in computer vision, i.e., the convolutional neural network (CNN), was used to map the association between PEFR and environmental data. The proposed model conveyed an MSE of 2.42 for the population-based and 1.36 for the individual-based analysis. Yahyaoui A. et al. [26] considered a lung disease, i.e., pneumonia and asthma, with 212 samples for a classification task. They commenced a private dataset from a Turkish hospital with 100 healthy, 64 pneumonia, and 48 asthma patients. Their input set consisted of 38 features, including clinical symptoms and lab parameters. They applied K-nearest neighbors (KNN), a method based on finding the closest neighbors in the features space and deep neural network (DNN), a model consisting of multiple layers in extracting complex features, to the data. Results depicted similar performance of 95% and 94.3% accuracy on both KNN and DNN classifiers.

A study by Makrufardi F. et al. showed the impact of extreme weather with 1.10-fold for symptoms and 1.18-fold disease event risk. They also found that the weather increases risk by 1.19 and 1.29 fold in children and females, respectively [27,28]. Intensive atmosphere, i.e., sulfur dioxide (*SO*_2_), carbon monoxide (CO), ozone (*O*_3_), nitrogen dioxide (*NO*_2_), particulate matter with a diameter of 10 micrometers or less (*PM*_10_), particulate matter with a diameter of 2.5 micrometers or less (*PM*_2.5_), weather and air pollution have a significant effect on asthma outbreak. A range of metrics exist that estimate the error among predicted and actual labels such as mean absolute error (MAE), mean squared error (MSE), and mean absolute percentage error (MAPE). MAE measures the average error, which is robust in the outliers, while MSE penalizes larger errors than MAE and is sensitive to the outliers. MAPE also provides the error rate but in terms of the percentage as shown in Eq 2 [29].

$$MAPE=\frac{1}{n}\sum_{i=1}^{n}\left|\frac{y_{i}-\tilde{y_{i}}}{y_{i}}\right|\times 100$$ (2)

$$Pearson\;Correlation=\sqrt{\frac{\sum_{i=1}^{n}(y_{i}-y_{avg}{)}^{2}-\sum_{i=1}^{n}(y_{i}-\tilde{y_{i}}{)}^{2}}{\sum_{i=1}^{n}(y_{i}-y_{\text{mean}}{)}^{2}}}$$ (3)

Where *y*_*i*_ and $\tilde{y_{i}}$ depicts actual and modeled *i*^*th*^ values, $y_{\text{mean}}$ is the average of actual values. The Pearson correlation coefficient shown in Eq 3 [29] was considered by Sung et al. [30] on weather and disease data released by the Korean government. The authors also predicted asthma occurrence using the DNN model, which showed 694.5 MAE, 920.06 MSE, and 11.72 MAPE error rates. A similar study on Korean children was carried out by Woo J. et al. [31] in which they forecasted the next day’s PEFR value using indoor air quality data, i.e., *PM*2.5, temperature, humidity, and carbon dioxide (*CO*_2_) levels for the previous day. The dataset consisted of four months of relevant data and was assessed using DNN and feedback-oriented recurrent neural networks, which execute feedback and attention mechanisms. The models were examined using 19 individuals’ data, which showed an average root MSE of 42.5 and MAPE of 14.0 for all subjects. Joe G. et al. implemented a classification-oriented task on the EHR data of asthma patients into three categories: non-severe exacerbation, emergency department visits, and hospitalization. They evaluated three ML models: LGR, RF, and gradient boosting decision tree. The study results showed that gradient boosting outperformed with the receiver operating characteristic curves of 0.71, 0.88, and 0.85, respectively [32].

The significance of ML algorithms in childhood asthma prediction was reviewed by Patel D. et al. [33] using a thorough literature review. The analysis revealed that ML algorithms can predict asthma more precisely than conventional models. Extreme Gradient Boosting (XGBoost) is a popular ML model that uses ensemble learning and gradient-boosting techniques to improve performance. Likewise, a support vector machine (SVM) is a classification and regression-based ML model, draws a hyperplane on the feature space that can differentiate the classes accurately. A comparison-based study on three ML models, i.e., XGBoost, SVM, and LGR, along with clinical rules, was conducted by Hond A. et al. [34] in which time series predictions were made using PEFR and asthma symptoms data. The severe risk was estimated using the XGBoost model. However, the prevailing performance for the LGR model was better, with an AUC of 0.88, than 0.85 for XGBoost. A review by Tanvi O. et al. [35] highlighted the importance of the XGBoost classifier in asthma exacerbation prediction. One of the published outcomes by the current study authors [36,37] used the same dataset to measure the effect of indoor air quality on PEFR. Since the dataset was imbalanced, up and over-sampling techniques were used. A transfer learning technique was tested to fine-tune the pre-trained model on new data with a similar domain knowledge having 1 and 2 hidden layers. The presented model improved the accuracy by 5.5-11.8%.

An association between outdoor weather and particulate matter was examined by Pyingkodi M. et al. [38] on PEFR values into two classes, i.e., safe and risk. The CNN, KNN, and SVM were considered for this objective. CNN was implemented to filter data features. The findings support the proposed methods for accurate asthma risk prediction. An innovative e-health application presented by Alharbi E. et al. [39] to predict asthma and safe route recommendations. The system can guide individuals to change the route based on asthma trigger risk for a specific area. Their study utilized a dataset of 665 records holding EHR, bio-signals, and environmental and spatial data to evaluate the XGBoost model. The study results demonstrated that the proposed system achieved 94% on ten asthma patients with 95.2% recall in recommended route generation. Seyed V. et al. [40] mapped asthma-prone areas considering weather factors such as climate and air pollution using the XGBoost model, achieving a 97% receiver operating characteristic curve. They also assumed explainable AI method, i.e., Shapley additive explanations that revealed spring & autumn rainfall and summer & winter temperature have a significant impact on asthma. Khasha R. et al. [41] organized 2870 records based on daily asthma assessments from 96 patients within 9 months. The data included clinical, medical history, and environmental variables to classify the time series data for a 7-day time window. Multiple ML models were used to experiment with the data, which revealed ML potential in asthma prediction. The efficacy of digital peak flow monitoring, which uses the DL model to predict next-day PEFR value, was assessed by Ananth S. et al. [42] on three months of data. The investigation outcomes showed it could expect the PEFR zone with 94±8.6% probability.

The existing work suggests a research gap in assessing a hybrid ML model for predicting PEFR for the following weeks. A comparable task was performed in [25,30,38] using ML and DL models without consideration of significant hybrid models. Likewise, in [31], PEFR was also predicted for future days based on pediatric asthma, which showed more work relevancy; however, DL models were evaluated, which comparatively exhausts more processing power. Therefore, the current study took an initiative to formulate a hybrid ML model, introducing novelty to a similar task. Another motivation for asthma prediction could be using physiological or bio-signals, performed in [21,39]; however, it requires signal data not currently available in our dataset.

## 3 Methodology

This study is designed to address the research gap outlined in Sect 2 by devising a hybrid model to forecast the following week’s PEFR values based on the given features. In addition, string matching and yesterday’s PEFR value were supplemented to the ML model input for more effective prediction. The variables such as age, gender, body mass index, yesterday’s PEFR, and indoor quality metrics, including temperature, humidity, *PM*_2.5_, and *CO*_2_ were considered for experiments.

### 3.1 Dataset for asthma analysis

An asthma patients dataset of 25 individuals having parameters mentioned above with age between 34 to 83 years, who collaborated with the ESCORT (Environmental health Smart study with COnnectivity and Remote sensing Technologies) study program [43] were logged between 28 December 2017 to 31 December 2018. All of them were consulted, counseled, and monitored by medical doctors and practitioners at Soonchunhyang University Bucheon Hospital, South Korea. The original study protocols related to the study were approved by the research ethics committee of Soonchunhyang University (IRB No. 1040875-201608-BR-030), and written informed consent was obtained from all participants. The participants of the study are non-smokers. Numerous features, including individual characteristics, PEFR, weather, and indoor and outdoor air quality, exist for each patient. The dataset has about 4000 records representing all patients in a ’comma-separated value’ format file. The missing values and outliers were addressed to prevent data inconsistency.

### 3.2 Inclusivity in global research

Additional information regarding the ethical, cultural, and scientific considerations specific to inclusivity in global research is included in the Supporting Information (SX Checklist).

### 3.3 String matching

String matching is a technique that compares the input variable with the given dataset and provides the reference PEFR of a specific record when the similarity index is high. To strengthen the suggested hybrid model at layer 2, an additional input in the form of a PEFR value was realized to close the gap between actual and predicted PEFR. Therefore, a string matching technique has been considered where the input features are compared row by row and column by column on the given dataset. As a result, a PEFR value called the “reference PEFR” of the relevant row has been fetched, where the matching results are high compared to the rest.

**Algorithm 1.** String Matching Algorithm



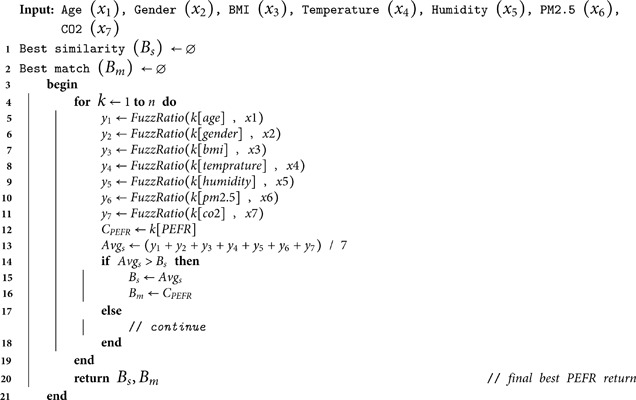



Algorithm 1 shows the predominant working of the proposed approach, which exploits fuzzy-wuzzy library [44] for the implementation. The library executes “Levenshtein Distance” to calculate the span among two variables and assigns a similarity value. First of all, the input variables are passed from *x*_*1*_ to *x*_*7*_ along side null (∅) assignment for best match (*B*_*s*_) and best PEFR (*B*_*m*_) attributes. Then, the “for loop” starts, which iterates on all dataset rows from 1 to n. The “fuzz ratio” function from the fuzzy-wuzzy library is revoked each time. It takes two parameters, including existing row/column data and input parameter, i.e., age, to calculate the similarity measure between them. The same procedure is repeated for all features from lines 5 to 11. At line 12, the relevant row *(k)* PEFR is fetched and stored into current PEFR (*C*_*PEFR*_). Once the current row operations are finalized, the algorithm finds the average resemblance outcome from all the variables, i.e., *y*_*1*_ to *y*_*7*_. An “if-else” condition is called, which decides whether the current similarity average $\left(Avg_{s}\right)$ is greater than the *B*_*s*_. If so, it replaces the existing values of *B*_*s*_ and *B*_*m*_, otherwise it continues with the loop. Once the *for loop* is finished, the values of *B*_*s*_ and *B*_*m*_ are returned.

### 3.4 Two-step approach and proposed model architecture

**Fig 1:**
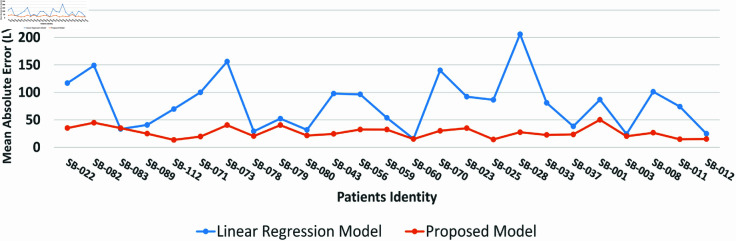
Proposed two-step approach that utilizes classification and regression model in addition to string matching and yesterday’s measurements to predict following weeks PEFR.

A two-step approach is proposed to find the best optimized PEFR value employing classification to regression tasks. First, the entire dataset is split into three percentiles; <50%, 50-80%, >80%, based on each patient’s PEFR value. Then, an ML model, i.e., RF, is considered to classify percentiles. The main reason to have three percentile zones split instead of two is that having single class for the 50-80% & >80% percentiles may be over-weighted over the second, i.e., <50%, resulting in more misclassification. Second, we hypothesized that the LR model would perform better once the relevant data is presented. Therefore, a two-layer regression model was implemented as shown in Fig 1. The classification model diverts the data according to the percentile cutoff. In addition, two distributed LR models are trained at layer 1, one for percentile >=50% and the second for <50% data in achieving the study hypothesis. Following the predicted classification label, input data is diverted to the relevant LR model out of the two demonstrated at layer 1.

Although the classification model provides three class results w.r.t. percentile range, 50-80%, and >80% percentiles cutoff are converged into one for further regression analysis because of the results interpretation, as shown in Table 4. It was hypothesized that the multiple trained LR models on grouped percentiles data, i.e., <50% and >=50%, return the best-predicted value than a single model because of the more relevant patterns in percentile-wise grouped training data. The classification was carried out to evaluate the hypothesis, and the final model was assessed based on original and predicted PEFR values.

The proposed model’s overall workflow is depicted in Fig 1. Once three PEFRs from various channels; the LR models, string matching, and yesterday monitoring, were realized at layer 1, we tested multiple approaches on them, i.e., average and weight assignment, to get the optimized single output value at layer 2, but none of the techniques produced the desired results. Therefore, we determined to place another LR model at the next layer 2, which was expected to improve the outcome. Nevertheless, further data was required for model training. Therefore, in data arrangement for this LR model at layer 2, the so far level algorithm, i.e., covering until layer 1, ran on random data of five patients several times concerning the collection of three PEFRs from the distributed LR models at layer 1, string matching, and yesterday’s measurement couples with the target variable as real PEFR. The training data for the LR model at layer 2 was designed so that all patients’ data should not appear together in any of the N-fold validation sets to avoid bias analysis. By taking this measure, we acquired roughly 2600 rows with minor repetition featuring the indicated columns for use in model training. It was ensured that the ultimate final analysis would remain unbiased due to obtaining and utilizing data from the original dataset in the following ways:

- The two hypothesized LR models at layer 1 produce slightly varied PEFR predictions with each execution, reducing the risk of identical values appearing in the final analysis.
- Yesterday’s measurements are utilized from the (predicted day - 1) PEFR without fetching the actual yesterday’s PEFR given in the dataset.
- It was realized that these five patients must not occur together in any of the N-fold sets. Thus, during performing the actual experiments, this generated training data is independent of the results in the context of already-seen data. The decision to choose RF and LR models is described in the section “results and discussion”.

**Algorithm 2.** Continuous PEFR Prediction Algorithm



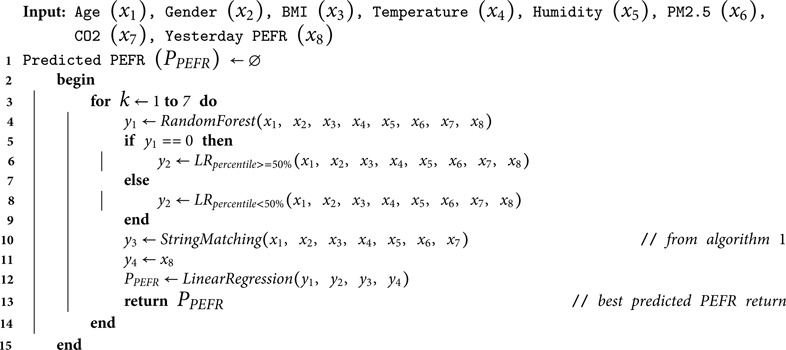



Algorithm 2 presents the step-wise procedure to predict the PEFR value for the following weeks. Yesterday PEFR value is supplemented with input features when calling the distributed LR model. It is worth noting that the PEFR recorded yesterday was initially captured solely from the dataset, and when predicting values for subsequent weeks, (predicted day - 1) value was called yesterday’s PEFR. The variables were normalized, except for yesterday’s PEFR, raising the concern that this non-normalized variable might dominate over the normalized variables and suppress the influence of environmental variables during prediction. To address this concern, the string matching technique presented in Algorithm 1 is introduced. This technique thoroughly examines and incorporates personal characteristics and indoor environmental variables, then retrieves the reference PEFR for the corresponding row in the ’comma-separated value’ format file with the highest similarity value, ensuring the dominance of the non-normalized variable is effectively mitigated. Then, this PEFR value aligned as an input in the final auxiliary LR model at line 12 of Algorithm 2. The loop follows the same procedure for days and following weeks by predicting the final PEFR each time.

## 4 Results and discussion

An experimental setup was established to evaluate the two-step model described in Sect 3. A jupyter notebook with TensorFlow, Sci-kit, Pandas, NumPy, MatplotLib, and Python as a programming language were deemed for experiments. The details are illustrated in the subsequent subsections.

### 4.1 N-fold cross validation

Evaluating the efficiency of the proposed algorithm on the entire dataset was concerned, with the data being divided into five sets for distribution. The K-fold cross-validation allows to assess the model efficiency on each patient’s data in terms of a test set. Therefore, we split the whole dataset into five-fold cross-validation, where 20 patients were in the training set, and the remaining 5 were part of the test set. The cross-validation was conducted this way so that each patient must become part of the test set at least once.

### 4.2 Metrics

It is crucial to choose the metrics carefully while assessing the proposed model evaluation. The dataset discussed earlier can be divided into two sections. The first includes patient characteristics, such as age, gender, and BMI, while the second contains weather and indoor air quality measures, such as temperature, humidity, *CO*_2_, and *PM*_2.5_. Therefore, a more suitable method to assess the proposed model’s performance, by calculating the error rate between actual and predicted outcomes, was chosen using mean absolute error (MAE) and relative absolute error (RAE) because these metrics provide a clear understanding of prediction accuracy, especially when dealing with mixed data types such as continuous patient characteristics and environmental measurements.

$$\text{MAE}=\frac{1}{n}\sum_{i=1}^{n}\left|y_{i}-{\hat{y}}_{i}\right|$$ (4)

MAE finds the mean absolute error between actual (*y*_*i*_) and predicted $\left({\hat{y}}_{i}\right)$ values. It does not rely upon the magnitude of the actual values but shows equal weights for all prediction errors. The unit of the MAE is considered equivalent to the problem domain that is being predicted, i.e., PEFR that has liters per minute and can be expressed as “L/min”. The implementation of the formula was acquired from the Sci-kit learn library [45,46].

$$\text{RAE}=\frac{\sum_{i=1}^{n}\left|y_{i}-{\hat{y}}_{i}\right|}{\sum_{i=1}^{n}\left|y_{i}-\bar{y}\right|}$$ (5)

RAE is the extended but normalized version of the MAE that concentrates on the relative to mean absolute deviation of MAE for the actual values. The *n* denotes number of observations, *y*_*i*_ is actual value for *i* observation, ${\hat{y}}_{i}$ is predicted value for *i* observation and $\bar{y}$ denotes the mean of actual values. The RAE can be expressed in percentages for easy and reliable interpretation. For example, RAE of 10% shows that the predicted values have an average absolute error of 10% of the mean actual values [47]. Both metrics are reliable in evaluating regression models, depicting that the less error, the higher the algorithm’s performance.

### 4.3 Classification models

The ML classification models, i.e., RF, SVM, were considered and evaluated to choose the reliable LR model at layer 1 of the presented architecture in Fig 1 through the enhanced classification results. The dataset was split into three percentiles, i.e., <50%, 50-80%, and >80%, based on given PEFR cutoff [48]. Each patient and his everyday history consist of input variables including age, gender, BMI, temperature, humidity, *PM*_2.5_, *CO*_2_, yesterday PEFR, and today PEFR was maintained. When the patient was exposed to a specific environment with some individual characteristics, his PEFR value was measured for that particular day, given in the dataset. To perform the classification, static labels, i.e., 0, 1, and 2, must be realized rather than quantitative measurements, i.e., PEFR. Therefore, today PEFR values were mapped into three percentile zones, as noted above. Once the percentile values were mapped, ’today PEFR’ was excluded from the input features for classification since we intended to find this value on unseen patients’ data by the LR model. Neither percentile nor ’today PEFR’ were part of the input features in both classification and regression. It was desired to be predicted by the ML model.

Subsequently, four ML models, including SVM, RF, decision tree, and gradient boosting, were selected for classification based on the mentioned percentile zones. The dataset was split into 80% and 20% ratios for training and testing, respectively, and the classification was carried out on population-based data. The primary motive of the classification was to predict and divert the data into relevant distributed LR models (out of two) for enhanced forecasting that enables bridging reliable association among similar grouped data.

Table 1 indicates the performance of four different ML classifiers, in which RF and gradient boosting outperform decision tree and SVM. The analysis were carried out on 80% and 20% data split to find out which ML model is more appropriate for the final five-fold cross-validation data evaluation, other than that Table 1 results have no influence on the final model performance. Therefore, we built a classifier for further 5-fold cross-validation tasks using RF. Since the model performed sufficiently for three class problems, it was realized not to consider any data enhancement approach at this stage. Similarly, in each cross-validation set, it was ensured that the test set included the complete dataset or history for each patient. For instance, if five patients are selected for the test set, his all recordings from each patient must be included in the test set for the individual-based analysis.

**Table 1:** ML classifiers performance in predicting percentile zones of asthma patients.

Model	Accuracy	Precision	*F*_1_ Score
Decision Tree	89.74%	90.48%	90.09%
Support Vector Machine	92.09%	91.46%	91.74%
Random Forest	93.45%	93.25%	93.34%
Gradient Boosting	93.08%	93.41%	93.22%

Table 2 depicts the in-depth evaluation of the RF classifier on the complete dataset, which indicated identical performance similar to Table 1. Each N-fold set’s trained model (into format .pkl) was saved and utilized for percentile zone prediction on a test set dedicated during the cross-validation.

**Table 2:** Evaluation of the RF classifier’s on five-fold cross validation.

N-Fold Validation	Accuracy	Precision	*F*_1_ Score
Cross Validation 1	91.92%	91.60%	91.74%
Cross Validation 2	93.15%	93.45%	92.78%
Cross Validation 3	91.48%	91.70%	91.22%
Cross Validation 4	93.63%	93.58%	93.57%
Cross Validation 5	94.51%	94.19%	94.32%

### 4.4 Regression models

Similar to the classification methods, it was necessary to determine the suitable regression model for improved performance. Therefore, initial regression analysis were realized for prediction tasks, where specific features are provided, and a model is needed to predict the PEFR value for the upcoming weeks. The proposed work exploits the LR model twice at layers 1 & 2 to forecast the required PEFR. However, the choice of the LR model entirely depends upon preliminary results given in Table 3 under 1^*st*^ cross-validation set.

**Table 3:** Performance comparison of four regression models on unseen patient data under MAE (L/min) and RAE (%) evaluation metrics.

Model	SB-022		SB-082		SB-083		SB-089		SB-112		avg MAE	avg RAE
	MAE	RAE	MAE	RAE	MAE	RAE	MAE	RAE	MAE	RAE		
**LR**	121.5	118.02	155.3	104.53	16.73	13.70	47.92	35.69	70.18	90.98	82.326 L/min	72.58%
**Polynomial**	93.42	183.34	264.34	298.45	20.87	17.37	44.21	46.89	76.58	191.45	99.884 L/min	147.50%
**Lasso**	84.89	35.67	243.55	157.85	26.55	16.55	29.07	39.13	75.55	188.87	91.922 L/min	87.61%
**Ridge**	78.55	99.40	245.17	153.23	42.53	17.51	28.16	37.90	68.15	103.10	92.512 L/min	82.23%

The LR model demonstrates an association among input features & target variables and minimizes error with the help of linear equation. It utilizes the learned coefficient from the linear equation to predict the value. Polynomial is an extended version of LR in terms of cubic or quadratic equations and is considered helpful for establishing non-linear connections among the data. Likewise, the Lasso model introduces a penalty of L1 regularization to shrink a few coefficients to zero and is valuable for feature selection. Ridge also uses an identical penalty with L2 regularization to prevent significant coefficients, which is practical for multicollinearity. Table 3 shows the MAE and RAE for 1^*st*^ cross-validation set with five unseen patients’ entire record sets. Since Table 3 expressed that the LR model outperformed others. Therefore, it was considered for further evaluation alongside the proposed algorithm. The headings starting with “SB” are the patient’s identification number to manage the privacy concern.

### 4.5 Percentile zones

The results given in Table 3 clearly suggest choosing the LR model showing lower average MAE & RAE, and therefore, we decided to evaluate the LR model further in reliable selection of the percentile zones. Nevertheless, deciding which cutoff would be smoother for the predictions was difficult. Therefore, we intended to formulate two scenarios, i) <80% and >=80% ii) <50% and >=50%, assuming from our prior experience. The experimental outcome depicted in Table 4 evidently suggests considering the percentile group based on lower MAE and RAE.

**Table 4:** Assessment of percentile zones for LR model at layer 1 calibration.

Percentile	SB-023		SB-025		SB-028		SB-033		SB-037		avg MAE	avg RAE
	MAE	RAE	MAE	RAE	MAE	RAE	MAE	RAE	MAE	RAE		
**<80% & >=80%**	60.22	0.96	17.39	1.27	29.53	1.23	26.92	5.17	22.52	0.88	31.32 L/min	1.90%
**<50% & >=50%**	34.76	0.55	14.24	1.04	27.43	1.14	22.41	4.30	23.44	0.92	24.46 L/min	1.59%

A slightly lower MAE and RAE favor the percentile group with a cutoff of <50% and >=50%. As a result, the alternative cutoff was not considered for further experiments.

### 4.6 Regression analysis and PEFR forecasting

The discussion in Sects 4.3 and 4.4 clarify the selection of RF and LR models, considering their performance and identifying appropriate percentile cutoff. Therefore, the architecture shown in Fig 1 was followed to execute the PEFR analysis on 5-fold cross-validation data. Since the dataset consisted of about on average 2-3 months tracking history of each patient, we chose sequential record sets for each patient, followed by cross-validation and testing sets. The existing version of the standalone LR model available at the Sci-kit learn library was also evaluated on the given dataset compared to the proposed model. Although a few studies, such as [31], were identified as relevant during the literature review due to their somewhat similar methodologies to the presented work, a direct comparison of results would be inappropriate. This is because of differences in the models used, the focus on childhood asthma versus adult asthma, and variations in datasets and input features.

The proposed model was used to generate and compare PEFR predictions across the entire dataset for each individual with unseen testing sets. One can observe that the MAE is comparatively higher in PEFR actual and predictions for most patients by the existing standalone LR model than the proposed model. Likewise, the proposed model shows a lower MAE, i.e., less than 50 accross each individual. A few patients, SB-083, SB-078, SB-079, SB-080, SB-060, SB-003, SB-037, and SB-012, showed somehow equal MAE for both of the models; the possible reason could be the complexity, i.e., abrupt PEFR shift in data patterns that were not well interpreted by the presented model.

The RAE was also estimated to show the performance of the proposed and standalone LR models for PEFR forecasting at each following day. The average was calculated based on the original and predicted PEFR for each patient separately to depict in a single plot. A lower RAE can be observed in most patients, with few having comparable w.r.t. single detached LR model. The RAE of SB-071 and SB-033 shown to be slightly higher because few datasets were comparatively more complex than others, possibly due to patients with other aligned medical conditions disturbing stable asthma.

Predictions made by the proposed model on the SB-008 dataset revealed a closer forecasting line w.r.t. actual values given in Fig 4. It is also possible that the original data may have a sudden drop in PEFR, i.e., shown as an extended negative spikes in the figure near day 81. The algorithm can still predict an equal value similar to the original. However, there is yet about a 100-points difference due to an unexpected change in the original data. The standalone LR model written as “Basic RM” cannot find this rapid drop. The additional reference PEFR provided through string matching and yesterday’s PEFR, along with LR projections at layer 1, enabled the supplementary LR model at layer 2 to capture the immediate change.

**Fig 2:**
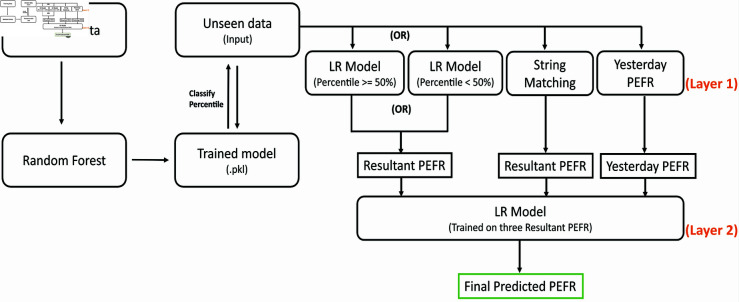
The MAE comparison between the proposed model, represented by the orange line, and standalone LR model, shown by the blue line, is presented across the entire dataset for each patient.

**Fig 3:**
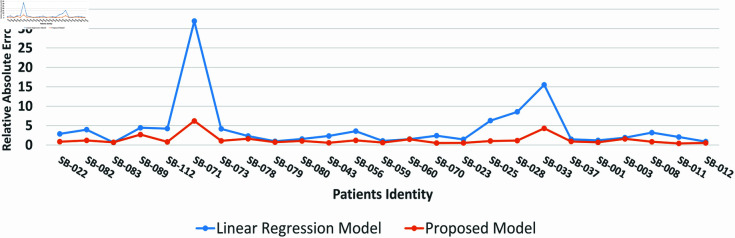
The RAE comparison between the proposed and standalone LR model for PEFR forecasting on unseen patient datasets.

**Fig 4:**
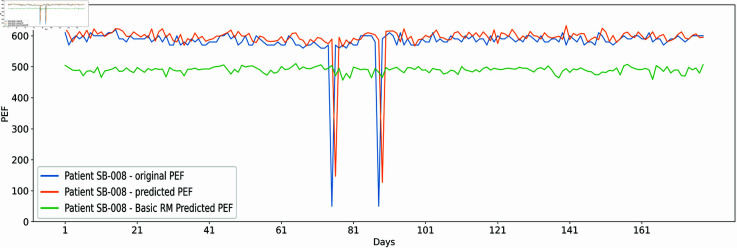
The x-axis shows PEFR (L/min) prediction results by the proposed model in the orange line and the standalone LR model (Basic RM) in the green line w.r.t. actual value in the blue line for patient SB-008 dataset at 178 days on the y-axis.

**Fig 5:**
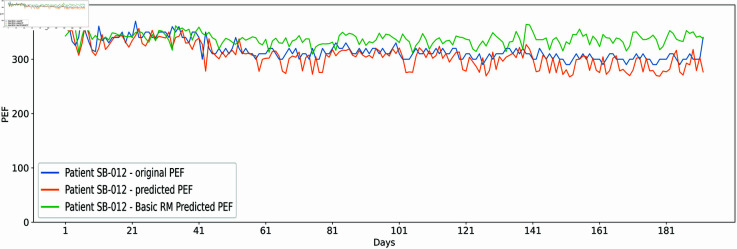
The x-axis represents PEFR (L/min) forecasting by the proposed model and the standalone LR model (Basic RM), aligned with the actual dataset values, while the y-axis indicates the patient SB-012 dataset over 192 days.

Likewise, the proposed approach also maintained equal performance on individual patient dataset SB-012 with closer predictions w.r.t. actual values. At the initial 70 days, the detached single LR also had well-aligned predictions. However, a clear gap can be observed throughout the dataset for the rest of the days. The outcome shows that two distributed trained LR models at layer 1 can uncover the association among data in a better way than the standalone LR model. The individual PEFR forecasting results for entire datasets, as shown in Figs 4 and 5 were estimated for each patient and exhibited similar outcomes. However, due to space constraints, it is not feasible to present all individual results. Therefore, Figs 2 and 3 provide a summarized overview of the results for all patients.

To highlight the overall performance of the proposed model, the average MAE and RAE were calculated for each patient using the 5-fold cross-validation technique, as shown in Table 5. The final average results demonstrate that the proposed model outperformed the standalone LR model, achieving lower MAE and RAE values of 27.064 L/min and 1.34%, respectively. The standalone LR model MAE & RAE is 2–3 times higher than the suggested work outcome, revealing its efficacy in forecasting PEFR values for asthma patients.

**Table 5:** Five-fold cross-validation results for proposed and standalone LR model.

Models	CV 1		CV 2		CV 3		CV 4		CV 5		avg MAE	avg RAE
	MAE	RAE	MAE	RAE	MAE	RAE	MAE	RAE	MAE	RAE		
**Linear Regression**	81.88	3.23	73.776	8.19	80.564	2.18	100.636	6.66	62.116	1.85	79.7944 L/min	4.42%
**Proposed Model**	30.526	1.25	28.346	2.14	26.772	0.89	24.456	1.59	25.224	0.82	27.0648 L/min	1.34%

Cross-validation is one of the suitable methods for tuning the hyperparameters and avoiding model overfitting [49,50]. The standalone LR and proposed hybrid model were tested on a k-fold cross-validation method where k=5 to ensure the model is balanced regarding low training and testing error. The test set was entirely unseen for the models during evaluation, depicting similar performance. A lower average MAE and RAE error along with Figs 2 and 3 showing error rate at all of the patients clearly indicate that the model performs best on testing sets without model overfitting. In addition, Figs 4 and 5 depict the proposed and standalone LR model predictions on unseen data that are compared with the actual data, thus, showing a closer gap between actual and proposed model predicted outcome supporting that the model is not overfitted. Similar results are available on the GitHub repository for all patients shared under the “availability statement”.

The proposed algorithm includes various components with partial dependencies on the previous element. For instance, if the RF model predicts the wrong classification, the rest of the element’s functionality will not be affected much. As discussed earlier, the classification model divides the data based on features, supporting the choice of a more reliable LR model given at layer 1. Then, these models uncover a more accurate association among the data for enhanced forecasting than a single detached LR model. Conversely, string matching closely analyzes the patient characteristics and indoor quality features to provide a reference PEFR to the supplementary LR model at layer 2 alongside yesterday’s PEFR. Although the outcome of the proposed model outperformed the standalone LR model, there is still a gap in assessing the model w.r.t. more diverse data. This includes asthma recordings from patients residing in various locations with differing environmental factors, potentially introducing more complex patterns within the data.

## 5 Conclusion

This study presented a hybrid approach for the prediction of peak expiratory flow rate (PEFR) of asthma patients. Our method combined ML models, featuring classification using RF and regression analysis with multiple LR methods. The RF model was utilized to classify the data for choosing a suitable distributed LR model at layer 1. The reliable distributed LR model trained on percentile zones separately, i.e., <50% and >=50%, established adequate associations among input data and PEFR predictions as apposed to a single standalone. In addition, a string-matching algorithm and yesterday’s PEFR were integrated to provide the baseline PEFR. The outcome of the three methods LR, similarity matching, and previous day PEFR, were input to the supplementary LR model at layer 2 for the final predictions. The model was evaluated on a dataset of 25 patients, each having on average 2-3 months recording enriched with personal attributes and indoor quality measures. The results depicted the lowest mean and relative absolute error of 27.064 L/min and 1.34%, respectively, indicating that the proposed model outperformed other existing alternatives. The study’s limitations include a relatively small dataset size and the uniformity of participants, who were predominantly from the same geographical area. This may introduce bias into the dataset’s features during analysis. In future work, we aim to analyze a larger dataset with more diverse participants. Additionally, physiological and biomedical signals identified during the review will be investigated for their potential in asthma prediction.

## Supporting information

SX Check List Additional information regarding the ethical, cultural, and scientific considerations specific to inclusivity in global research. (DOCX)
